# The Indirect Association of Resilience in the Relationship Between Educational Support and Transition Status Among Novice Home Healthcare Nurses

**DOI:** 10.1002/nop2.70849

**Published:** 2026-09-22

**Authors:** Shaimaa Mohamed Amin, Heba Emad El‐Gazar, Mohamed Ali Zoromba, Heba Sobhy Mohamed, Mohammed Adel Abd Elhafeez Elbakry, Mohamed Ahmed Aly Mohamed, Mohamed Hussein Ramadan Atta, Sally Mohammed Farghaly Abdelaliem, Nadiah A. Baghdadi, Ayman Mohamed El‐Ashry

**Affiliations:** ^1^ Department of Community, Psychiatric, and Mental Health Nursing, College of Nursing Qassim University Buraydah Saudi Arabia; ^2^ Community Health Nursing Department, Faculty of Nursing Damanhour University Damanhour Egypt; ^3^ Nursing Services Department Qassim University Medical City Buraydah Saudi Arabia; ^4^ Department of Nursing Administration and Education, College of Nursing Imam Mohammad Ibn Saud Islamic University (IMSIU) Riyadh Saudi Arabia; ^5^ College of Nursing Prince Sattam bin Abdualziz University Al‐Kharj Saudi Arabia; ^6^ Faculty of Nursing Mansoura University Mansoura Egypt; ^7^ Nursing Administration, Faculty of Nursing Zagazig University Zagazig Egypt; ^8^ Nursing Department College of Applied Medical Sciences, Prince Sattam Bin Abdulaziz University Wadi Addawasir Saudi Arabia; ^9^ Psychiatric and Mental Health Nursing, Faculty of Nursing Alexandria University Alexandria Egypt; ^10^ Department of Nursing, Faculty of Allied Health Sciences Kuwait University Kuwait Kuwait; ^11^ Department of Nursing Administration, Faculty of Nursing Alexandria University Alexandria Egypt; ^12^ Department of Nursing Management and Education, College of Nursing Princess Nourah bint Abdulrahman University Riyadh Saudi Arabia

**Keywords:** educational support, home healthcare, novice nurses, nursing adaptation, professional integration, resilience, transition status

## Abstract

**Aim:**

To examine the association between educational support and transition status among novice home healthcare nurses and to assess whether resilience shows an indirect association in this relationship.

**Design:**

A descriptive, cross‐sectional, correlational study was conducted with 215 novice nurses employed in home healthcare agencies.

**Methods:**

Data were collected using two standardized English instruments: the Educational Support Assessment Scale, the Transition Status Scale for Newly Graduated Nurses and the Arabic version of the Connor–Davidson Resilience Scale. Two instruments were translated into Arabic and culturally adapted for this study through forward–backward translation, expert panel review and pilot testing. Internal consistency and confirmatory factor analysis were used to examine reliability and preliminary construct validity. Descriptive statistics, Pearson correlations, linear regression and structural equation modelling were used to analyse the data.

**Results:**

Participants reported moderate to high educational support, high resilience and generally positive transition status. Active coping strategies and interpersonal integration were the highest‐rated transition dimensions, whereas work–life balance was the lowest. Educational support was positively associated with resilience and transition status. Resilience also showed a statistically significant indirect association between educational support and transition status. Because data were collected at one time point, this indirect pathway should be interpreted as an associative statistical model rather than evidence of causal mediation.

**Conclusions:**

Educational support, resilience and transition status were positively associated among novice home healthcare nurses. Resilience may be an important psychological factor linked to the relationship between educational support and transition status. Longitudinal studies are needed to confirm the temporal and causal nature of these associations.

**Implications for the Profession and/or Patient Care:**

Home healthcare agencies should provide structured educational support that combines clinical skill development with resilience‐building strategies, including guided reflection, mentoring, stress‐management training and peer support.

**Patient or Public Contribution:**

None.

## 1 Introduction

The shift to practice from education is a pivotal time for novice nurses. A novice nurse is usually a nurse who is a recent graduate with less than 2 years of experience (Arrowsmith et al. 2016). During this time, nurses should transition from supervised learning to independently performing clinical tasks, build confidence in their practice, build an understanding of their role in the work environment, and manage the emotional and practical challenges of patient care (Graf et al. 2020; Ma et al. 2021). This is a crucial transition as adverse adjustment may be associated with transition shock, burnout, decreased job satisfaction, desire to leave the profession and potential threats to quality of care and patient safety (Ma et al. 2024; Asseiri et al. 2025).

The concept of transition status illustrates how a new nurse adapts and integrates into the work environment when entering their profession (Ma et al. 2021). The transition status consists of 5 dimensions: Interpersonal integration, Active coping strategies, Profession‐related positive emotions, Competence for Nursing Work, Work–life balance (Ma et al. 2021). The dimensions suggest that effective transition goes beyond technical proficiency. It also entails social integration, emotional adaptation, coping ability and the ability to sustain a work‐life relationship.

The processes of transition have been frequently studied in hospital settings, but may be unique to novice nurses in home healthcare settings. Home healthcare nurses provide care in patients' homes, instead of the more structured setting of institutions. This means that the children need to be able to make choices early, have some flexibility in decision making, communicate with patients and family members and manage multiple homes and care needs with less direct support from peers and/or supervisors. Thus, the process of developing from novice HCHC nurses to advanced practice nurses could be especially sensitive to the accessibility of organized educational support and individual nurses' psychological adaptation.

This process of novicing can be viewed in the context of the larger organizational body of research on the process of adjusting to and adapting to the workplace. In every occupation, adaptation to the work context includes learning the expectations for the role, building competence and self‐efficacy, gaining social acceptance, adopting coping mechanisms and becoming acculturated to the workplace (Bauer et al. 2007; Saks and Gruman 2018). In this view, the transition status of novice nurses represents a type of workplace adaptation specific to the nursing profession. It embodies the relationship between the resources of the organization, the support received from social networks, learning the new role and the individual's own resources needed to cope in the new or challenging work environment.

Organizational resources that can help with this adaptation are the provision of educational support. In HHC nursing, educational support involves the planned provision of learning experiences for novice nurses to gain new knowledge, experience, reflect on practice and receive guidance from the management of HHC (Mori et al. 2023). Such support can help novice nurses develop their understanding of their nurse role, build confidence and guide decision‐making in managing clinical uncertainty. The importance of structured support, preceptorship, learning opportunities and organizational guidance for newly graduated nurses' transition to practice has been highlighted in previous studies (Arrowsmith et al. 2016; Graf et al. 2020; Baharum et al. 2023; Ma et al. 2024). Yet, the role of educational support for novice home healthcare nurses in the transition status has not been explored enough.

Another thing that could play a role is resilience. Resilience is commonly defined as being able to function effectively or recover from stress, uncertainty and adversity (Southwick et al. 2014). In occupational research, resilience is considered a personal adaptive resource for coping with job demands and maintaining effective functioning under stress (Robertson et al. 2015). It is also seen as a part of positive psychological capital that, alongside hope, optimism and self‐efficacy, has been linked to employee well‐being and functioning in work (Luthans et al. 2007). Likewise, in the job demands–resources model, employee adjustment and positive work outcomes (PWO) are dependent upon both organizational resources and personal resources (Bakker and Demerouti 2007; Xanthopoulou et al. 2007).

In the nursing field, the concept of resilience has been linked to adaptation to professional demands, outcomes from transition and indicators of retention for new nurses (Meyer and Shatto 2018; Xin et al. 2024). Resilient novice nurses might better be able to cope with uncertainty and respond to stressful clinical situations in a positive manner, and gain confidence while gaining competence. This could be particularly significant in‐home health nursing, where newly trained nurses may work more independently and have less immediate help from peers during patient care. Resilience can therefore be used to explain the association of educational support with the transition status.

Although there is increasing research evidence on adaptation at work, transition to the nursing profession and resilience, there are two gaps in the literature. For one, the research on newly graduated nurses has been conducted within hospital/institutional environments with little attention paid to novice home health care nurses. Second, previous research has tended to focus on the direct link between resources in the workplace or school setting and transition outcomes, rather than the psychological processes by which resources might be linked to adjustment. The importance of addressing these gaps is that educational support may have a direct impact on transition status, mediated by knowledge and competence building, as well as an indirect impact through association with resilience as an adaptive psychological resource.

The purpose of this study was to investigate the relationship between educational support and transition status in novice home healthcare nurses and to determine if resilience has an indirect relationship in this relationship.

### 1.1 Research Question

What is the relationship between educational support and transition status among novice home healthcare nurses, and is resilience indirectly associated with this relationship?

### 1.2 Hypotheses

Higher educational support is positively associated with transition status.

Higher educational support is positively associated with resilience.

Resilience shows an indirect association in the relationship between educational support and transition status.

### 1.3 Study Design and Setting

The study employed a descriptive, cross‐sectional, correlational design and followed the Strengthening of the Reporting of Observational Studies in Epidemiology (STROBE) guidelines to ensure rigorous reporting standards (von Elm et al. 2007). The study was carried out in several home healthcare agencies in Sharqia Governorate in the northeastern part of Egypt in the Nile Delta region where the study was conducted. The need for community‐based care has been increasing in this area, particularly for those with chronic diseases, disabilities or post‐hospitalization support needs. The home healthcare providers in Egypt provide services in both urban and rural areas, and they face different challenges in staffing, infrastructure and access to services. New nurses working in these facilities need to quickly adapt to independent nursing practice and care for patients with complex care requirements in an environment of limited resources. This highlights the importance of educational support and resilience in facilitating a successful transition to work. Given the region's various healthcare needs, Sharqia is an interesting and relevant place to explore novice nurses' experiences.

### 1.4 Sample Size Calculation and Sampling

The sample size was determined a priori with the help of Epi Info 7 (StatCalc, CDC). As this was a cross‐sectional correlational design, the calculation was based on the ability to detect a small to moderate correlation (*r* = 0.20) between educational support and transition status. StatCalc suggested a minimum number of 194 participants for a two‐tailed test with α = 0.05 and 80% power. A sample of 222 novice home healthcare nurses was used to allow for potential non‐response and incomplete data (~10%). Of those, 7 questionnaires were excluded since they were incomplete, and 215 questionnaires were used for the regression and structural equation modelling analyses (Centers for Disease Control and Prevention 2020; Cohen 1992; Kline 2016).

Given the usage of CFA and a modified indirect‐association model, the final sample's adequacy was evaluated in proportion to the complexity of these analyses. Sample‐size requirements for CFA and SEM are model‐dependent and vary depending on factors such as the number of indicators and factors, magnitude of factor loadings and structural paths and estimation method; thus, fixed sample‐size rules were not used as the sole criterion for adequacy (Wolf et al. 2013).

The final sample of 215 enabled the estimate of the specified CFA models using DWLS for ordinal indicators, with all models converging without unacceptable solutions. Nonetheless, because the ESA‐NHHN and TSSNGNs constituted rather large second‐order CFA models, their measurement‐model results were taken with caution. In the indirect‐association study, published simulation data indicates that roughly 71 individuals are necessary to reach 80% power for a bias‐corrected bootstrap test when both component routes are of medium magnitude (Fritz and MacKinnon 2007). Thus, the final sample of 215 exceeded this threshold, providing additional evidence for the sample's suitability for estimating the adjusted indirect‐association model. However, the initial a priori sample‐size estimate was based on the principal correlational goal rather than a model‐specific Monte Carlo power study.

The participation of the subjects was done by a convenience sampling method, which is the selection of the type of subjects who are available during the time of data collection and are eligible for the study. This method was feasible for novices working in different home healthcare agencies, but could restrict the representativeness of the sample. Furthermore, no data were collected to measure the rate of response within each agency participating. Thus, it was not possible to assess if there were any institutional‐level differences in participation, which could suggest selection bias.

The inclusion criteria were home healthcare nurses who were in their first year of working in home healthcare agencies in Sharqia Governorate, and who had received any type of home healthcare educational support or orientation. Participants had to be registered nurses and have given informed consent. The exclusion criteria were nurses with more than 2 years of experience in home healthcare, hospital‐based care/institutional care nurses who were not in community‐based home care and nurses who were on extended leave during data collection. Further, those who had already completed resilience or transition‐specific training in outside programmes not part of their agency's education programme were not included to ensure consistency of educational support.

## 2 Instruments

### 2.1 The Demographic Form

A structured sociodemographic form was used to collect background information from the participants. Some of the most important variables included in this form were age, sex, place of residence, family income and marital status. It also collected professional data such as years of experience in home healthcare, educational level, previous special training in home health care, average number of patients seen per week and average hours worked per week. Furthermore, information was gathered on the kind of education provided and the type of employer, a home health care agency.

### 2.2 The Arabic Version of Connor–Davidson Resilience Scale (10‐ Item CD‐RISC)

Resilience was assessed using the 10‐item Connor–Davidson Resilience Scale (CD‐RISC‐10), developed by Campbell‐Sills and Stein (2007) as a brief version of the original Connor–Davidson Resilience Scale (Connor and Davidson 2003). In the current study, we used the Arabic version that was adapted and translated by Bizri et al. (2022). The scale measures the ability to cope with and recover from adversity. Each item is rated on a 5‐point Likert scale ranging from 0 (“Not true at all”) to 4 (“True nearly all the time”), with higher total scores indicating greater resilience. The CD‐RISC‐10 is commonly used as a unidimensional measure of resilience and has demonstrated good psychometric properties, including high internal consistency (Cronbach's α = 0.85–0.92), good test–retest reliability (approximately 0.87) and satisfactory construct validity. In the present study, the Arabic‐translated version demonstrated excellent internal consistency (Cronbach's *α* = 0.91).

### 2.3 The Educational Support Assessment Scale of Novice Home Healthcare Nurses (ESA‐NHHN)

Mori et al. (2023) have developed the Educational Support Assessment Scale, which is a validated instrument to assess the level of educational support felt by novice home healthcare nurses. The scale consists of 4 subscales: acquiring new knowledge (12 items), concrete experience (10 items), reflective observation of experience (6 items) and support by home healthcare agency managers (6 items). Responses are scored on a 5‐point Likert scale from 1 (strongly disagree) to 5 (strongly agree), with the overall score indicating the level of perceived educational support.

In the present study, confirmatory factor analysis (CFA) was performed to evaluate the original four‐factor structure of the ESA‐NHHN and its representation by a higher‐order Educational Support construct. The second‐order CFA demonstrated acceptable model fit (scaled CFI = 0.949, scaled TLI = 0.945, scaled RMSEA = 0.079, SRMR = 0.059), with strong standardized loadings of the four first‐order dimensions on the higher‐order Educational Support factor (*β* = 0.83–0.98), supporting the use of the overall total score in the subsequent analyses (see Data S1).

Although the scale comprises four related dimensions, it also yields an overall total score reflecting the respondent's overall perceived educational support. The original validation study supported the four‐factor structure using exploratory and confirmatory factor analyses and demonstrated good internal consistency (Cronbach's *α* = 0.84–0.95 across subscales) and test–retest reliability (ICC = 0.89). In the present study, the Arabic‐translated version demonstrated good internal consistency for the overall scale (Cronbach's α = 0.89).

### 2.4 The Newly Graduated Nurse Transition Status Scale (TSSNGNs)

The Transition Status Scale was designed and validated by Ma et al. (2021) to evaluate the transition experiences of newly graduated nurses in entering and adapting to clinical practice. The scale consists of 38 items, which are divided into five subscales: Interpersonal Integration (10 items), Active Coping Strategies (10 items), Profession‐Related Positive Emotions (seven items), Competence for Nursing Work (seven items) and Work–Life Balance (four items). The items are rated on a 5‐point Likert scale from strongly disagree (1) to strongly agree (5). Scores higher than 50 reflect successful adjustment to professional nursing roles. The original validation study demonstrated good psychometric properties, with support for the five‐factor structure through exploratory and confirmatory factor analyses, high overall internal consistency (Cronbach's *α* = 0.92), and good test–retest reliability (ICC = 0.87). In the present study, the Arabic‐translated version demonstrated good internal consistency for the overall scale (Cronbach's *α* = 0.90). In the present study, a second‐order confirmatory factor analysis was conducted to examine whether the five first‐order TSSNGNs dimensions could be represented by an overarching Transition Status construct. Using DWLS estimation for the ordinal items, the model showed marginal‐to‐acceptable fit: scaled χ^2^ (660) = 1720, *p* < 0.001, scaled CFI = 0.926, scaled TLI = 0.921, scaled RMSEA = 0.087, 95% CI [0.082, 0.092], and SRMR = 0.074. All five dimensions loaded significantly on the higher‐order factor (standardized *β* = 0.585–0.950), providing support for the use of the overall transition status score in the primary analyses (Data S1).

Although the Educational Support Assessment Scale and the Transition Status Scale comprise multiple related dimensions, both instruments provide overall total scores representing the broader constructs of perceived educational support and transition status, respectively. Consistent with the aim of the present study, which was to examine the overall associations among educational support, resilience, and transition status, overall composite scores were calculated for each of the three principal study variables. The second‐order CFA findings supported the representation of these dimensions by higher‐order constructs.

For the correlation, regression, and mediation analyses, one overall composite score was calculated for each of the three principal study variables: resilience, educational support, and transition status. These three total scores were analysed as observed continuous variables. The structural model did not include latent variables or a measurement model, and individual items or subscale scores were not used as indicators in the mediation analysis. The exploratory factor analyses were undertaken solely to give preliminary data about the structure of the Arabic‐translated instruments in the current sample.

## 3 Study Procedures

### 3.1 Tool Preparation & Pilot Study

Permission to use the ESA‐NHHN and TSSNGNs including permission to translate and culturally adapt the latter two was requested from the original authors/developers via email. No response was received. As two instruments are widely cited in the published literature and are generally accessible online for academic and research purposes, the research team proceeded with their use and adaptation as described below.

A structured translation and cultural adaptation process was conducted for the ESA‐NHHN and TSSNGNs. First, the original English versions of the scales were translated into Arabic by two independent bilingual translators fluent in both English and Arabic. One translator had a nursing/healthcare background to ensure conceptual accuracy, while the other had linguistic expertise to ensure clear and natural Arabic wording. The two Arabic translations were compared and reconciled into one agreed Arabic version by the research team.

Back‐translation was then performed by two independent bilingual translators who had not seen the original English versions. The back‐translated English versions were compared with the original tools to identify any differences in meaning. Any unclear or culturally inappropriate wording was discussed and revised until conceptual equivalence was achieved.

The Arabic versions were then reviewed by an expert panel consisting of two assistant professors in nursing, one lecturer in nursing education, one clinical nurse specialist in home healthcare and one bilingual language expert. The panel assessed each item for linguistic clarity, cultural relevance, content appropriateness and conceptual consistency with the original scales. Minor wording modifications were made according to their feedback to improve clarity and suitability for Arabic‐speaking novice home healthcare nurses.

A pilot test was conducted with 20 Arabic‐speaking home healthcare nurses who were like the target study population but were not included in the final sample. Participants completed the questionnaires and provided feedback on item clarity, wording, length and cultural suitability. Based on their comments, minor language modifications were made. No items were removed. Internal consistency was also assessed during the pilot phase to support the reliability of the translated instruments.

### 3.2 Data Collection

Data was collected over a three‐month period from novice home healthcare nurses employed across various healthcare settings. Ethical approval was obtained from the appropriate institutional review board, and informed consent was secured from all participants prior to participation. Participants were invited to complete an online self‐administered questionnaire, which consisted of demographic items along with three Arabic‐translated and culturally adapted versions of standardized instruments: the Connor‐Davidson Resilience Scale (CD‐RISC‐10), the Educational Support Assessment Scale (ESA‐NHHN) and the Transition Status Scale for Newly Graduated Nurses (TSSNGNs).

The questionnaire link was distributed electronically via email and professional communication platforms commonly used within the participating healthcare institutions. Clear instructions were provided at the beginning of the survey, and participants were able to contact the research team with any questions or concerns during the data collection period. To enhance response rates, reminder messages were sent periodically throughout the data collection window. All responses were anonymous, and data were securely stored to ensure confidentiality and integrity throughout the research process.

### 3.3 Ethical Considerations

Ethical approval for this study was obtained from the Research Ethics Committee of the Faculty of Nursing, Zagazig University, Egypt, under reference number (295). In addition, permission to conduct the study was secured by clinic directors following a clear explanation of the study's purpose. The research strictly followed the ethical guidelines of the Declaration of Helsinki to uphold participants' rights and well‐being. All participants provided written informed consent after being fully briefed on the study's objectives. Anonymity and privacy were diligently maintained, and all data were handled with strict confidentiality. Participants were also assured of their right to voluntarily withdraw from the study at any point without any negative repercussions.

### 3.4 Statistical Analysis

Data were analysed using IBM SPSS Statistics version 26, IBM SPSS AMOS version 26 and jamovi with the SEMLj module. Statistical significance was set at a two‐sided *p*‐value of < 0.05. Before the main analyses, the data were screened for completeness, values outside the theoretical ranges of the instruments, and univariate outliers. Univariate outliers were examined using standardized scores (|*z*| > 3.29) and boxplots. No extreme values requiring exclusion were identified.

Descriptive statistics, including frequencies, percentages, means, standard deviations and observed ranges, were used to summarize participants' sociodemographic and professional characteristics and the principal study variables. Internal consistency was assessed using Cronbach's alpha for the overall scores of the three instruments.

Confirmatory factor analysis was conducted to evaluate the measurement structure of the Arabic‐translated instruments and to determine whether their overall scores could be used in the principal analyses. Because the questionnaire items were measured on ordered 5‐point response scales, CFA models were estimated in jamovi using diagonally weighted least squares (DWLS) with robust standard errors. A one‐factor model was evaluated for CD‐RISC‐10. Second‐order CFA models were evaluated for the ESA‐NHHN and TSSNGNs, in which their respective first‐order dimensions loaded onto higher‐order Educational Support and Transition Status factors. Standardized factor loadings and model‐fit indices were reported. Indirect effects were estimated using a nonparametric bootstrap procedure with 5000 bootstrap resamples, and statistical significance was assessed using bias‐corrected (BC) 95% confidence intervals.

Model fit was evaluated using the chi‐square statistic (χ^2^), the χ^2^/df ratio, the Comparative Fit Index (CFI), the Tucker–Lewis Index (TLI), the Root Mean Square Error of Approximation (RMSEA) with its confidence interval and the Standardized Root Mean Square Residual (SRMR). CFI and TLI values of approximately 0.90 or higher were considered indicative of acceptable fit and values approaching 0.95 indicative of good fit. RMSEA values of approximately 0.08 or lower and SRMR values of 0.08 or lower were considered indicative of acceptable fit. These values were treated as interpretive guidelines rather than absolute decision rules, and the indices were evaluated collectively because their performance may vary according to model complexity, estimator and sample characteristics (Browne and Cudeck 1993; Hu and Bentler 1999).

After the measurement analyses, one overall composite score was calculated for educational support, resilience and transition status. Pearson's correlations were used to examine the unadjusted bivariate associations among these observed continuous variables. Partial correlations were then calculated while controlling for the selected sociodemographic and professional covariates. Linear regression analysis was used to examine the adjusted associations between the principal study variables.

The hypothesized indirect‐association model was subsequently examined in AMOS using the three overall composite scores as observed variables. Educational support was specified as the predictor, resilience as the intervening variable and transition status as the outcome. Sex, educational level, prior specialized training, average working hours per week and type of agency were included as covariates. Standardized direct, indirect and total effects, standard errors, *p*‐values and 95% confidence intervals were reported. The structural model did not include latent variables, individual items or subscale scores. Because the data were collected at one time point, the indirect pathway was interpreted as an associative statistical model rather than evidence of temporal or causal mediation.

## 4 Results

Table 1 presents the sociodemographic characteristics of 215 novice home healthcare nurses. The majority were above the age of 23 (91.63%), female (66.05%), lived in rural areas (55.35%), and reported having a sufficient income (64.65%). While age and residence made no significant differences in any of the three outcomes, sex and marital status were associated with notable variations. Male nurses showed considerably better resilience (*p* < 0.001) and transition status (*p* = 0.023) than females. Furthermore, resilience scores changed considerably depending on marital status (*p* = 0.025) with single nurses reporting the highest scores.

**TABLE 1:** Mean scores of educational support, resilience and transition status across Sociodemographic Characteristics of novice home healthcare nurses (*N* = 215).

Variable	*N* (%)	Educational support, mean ± SD	Resilience, mean ± SD	Transition status, mean ± SD
Age				
< 21	15 (6.98%)	142.67 ± 17.93	31.33 ± 6.66	156.27 ± 13.82
21–23	3 (1.4%)	132.00 ± 16.70	30.80 ± 5.84	160.33 ± 21.50
> 23	197 (91.63%)	132.40 ± 16.97	28.36 ± 6.18	148.57 ± 17.94
*p*‐value	—	0.081	0.251	0.151
Sex				
Male	73 (33.95%)	134.99 ± 19.94	31.47 ± 5.94	153.10 ± 18.75
Female	142 (66.05%)	132.14 ± 15.51	27.08 ± 5.77	147.31 ± 17.04
*p*‐value	—	0.250	< 0.001	0.023
Place of residence				
Rural	119 (55.35%)	132.85 ± 14.74	28.31 ± 5.78	148.96 ± 16.47
Urban	96 (44.65%)	133.43 ± 19.82	28.90 ± 6.65	149.67 ± 19.41
*p*‐value	—	0.806	0.505	0.772
Family income				
Not enough	48 (22.33%)	131.08 ± 19.93	26.93 ± 6.46	147.17 ± 21.83
Enough	139 (64.65%)	133.04 ± 16.54	29.12 ± 5.89	149.98 ± 16.22
Enough & save	28 (13.02%)	136.89 ± 14.80	28.67 ± 6.76	149.39 ± 18.13
*p‐*value	—	0.363	0.107	0.642
Marital status				
Single	67 (31.16%)	133.25 ± 21.07	30.00 ± 6.57	151.16 ± 18.06
Married	144 (66.98%)	132.83 ± 15.20	27.81 ± 5.88	148.01 ± 17.56
Widowed	3 (1.4%)	135.00 ± 1.00	29.67 ± 2.51	154.67 ± 6.51
Divorced	1 (0.47%)	—	—	—
*p‐*value	—	0.571	0.025	0.079

*Note:* Independent‐samples *t*‐test was used for two‐category variables, and one‐way ANOVA was used for variables with more than two categories. Statistical significance was set at *p* < 0.05.

Abbreviation: SD, standard deviation.

Table 2 describes the professional characteristics of novice home healthcare nurses. Nurses with 1–2 years of experience had the most educational support (140.88 ± 17.44), a statistically significant difference (*p* = 0.043). Diploma holders outperformed all three outcomes (*p* < 0.05), particularly in resilience and transition status. Those who have prior specialized training in home healthcare scored statistically significant higher in all variables (*p* = 0.002–0.003). Nurses who worked more than 60 h per week demonstrated substantially higher resilience (*p* = 0.029) and transition status (*p* = 0.019). Meanwhile, private agency nurses rated themselves considerably higher in resilience (*p* = 0.018) and transition status (*p* = 0.026) than those in public agencies.

**TABLE 2:** Mean scores of educational support, resilience and transition status across Professional Characteristics of novice home healthcare nurses (*N* = 215).

Variable	*N* (%)	Educational support, mean ± SD	Resilience, mean ± SD	Transition status, mean ± SD
Years of experience in home care				
< 6 mos	72 (33.5%)	131.09 ± 19.17	27.59 ± 6.74	147.95 ± 16.98
6 months–1 year	143 (66.5%)	134.11 ± 16.00	29.06 ± 5.83	149.94 ± 18.23
*p*‐value	—	0.187	0.181	0.420
Educational level				
Technical nursing diploma	54 (25.12%)	138.67 ± 16.56	31.46 ± 5.75	156.65 ± 16.80
Bachelor's degree	136 (63.26%)	131.51 ± 16.24	27.75 ± 6.05	147.87 ± 15.36
Master's degree	25 (11.63%)	129.80 ± 20.94	26.84 ± 5.96	141.00 ± 25.91
*p*‐value	—	0.019	< 0.001	< 0.001
Prior Specialized training in home healthcare				
Yes	91 (42.33%)	137.37 ± 18.28	30.00 ± 6.50	153.70 ± 17.53
No	124 (57.67%)	129.98 ± 15.62	27.53 ± 5.73	146.02 ± 17.36
*p*‐value	—	0.002	0.004	0.002
Average patient load per week				
1–5 pts.	110 (51.16%)	131.58 ± 18.43	28.47 ± 6.38	148.25 ± 17.58
6–10 pts	43 (20.0%)	133.49 ± 14.70	27.86 ± 6.08	149.00 ± 19.06
11–15 pts	15 (6.98%)	132.80 ± 20.06	28.93 ± 6.47	153.73 ± 23.61
> 15 pts	47 (21.86%)	136.43 ± 15.06	29.36 ± 5.76	150.49 ± 15.18
*p*‐value	—	0.450	0.704	0.678
Average working hours per week				
< 20 h	42 (19.53%)	129.36 ± 20.49	27.21 ± 6.78	142.24 ± 18.87
20–40 h	85 (39.53%)	132.58 ± 15.30	28.03 ± 5.53	150.01 ± 15.19
41–60 h	51 (23.72%)	133.53 ± 18.05	28.76 ± 6.33	150.06 ± 19.35
> 60 h	37 (17.21%)	138.00 ± 15.15	31.11 ± 6.15	154.49 ± 18.26
*p*‐value	—	0.162	0.029	0.019
Type of educational support				
Orientation program	73 (33.95%)	132.51 ± 18.65	28.21 ± 6.69	148.75 ± 18.66
Online learning modules	56 (26.05%)	135.55 ± 14.74	29.76 ± 5.75	150.23 ± 17.27
On‐the‐job training/workshops	63 (29.3%)	132.17 ± 16.58	28.16 ± 6.14	148.92 ± 16.99
Peer mentoring	23 (10.7%)	131.61 ± 19.58	27.96 ± 5.58	149.57 ± 19.54
*p*‐value	—	0.664	0.419	0.968
Type of home healthcare agency				
Public	142 (66.05%)	132.11 ± 16.01	27.87 ± 6.08	147.34 ± 17.61
Private	73 (33.95%)	135.05 ± 19.14	29.96 ± 6.17	153.04 ± 17.69
*p*‐value	—	0.233	0.018	0.026

*Note:* Independent‐samples *t*‐test was used for two‐category variables, and one‐way ANOVA was used for variables with more than two categories. Statistical significance was set at *p* < 0.05.

Abbreviation: SD, standard deviation.

The descriptive statistics for educational support, resilience and transition status among novice home healthcare nurses in Table 3. The mean educational support score was 133.11 (SD = 17.15), ranging from 55 to 170. The observed ranges for all variables fell within their theoretical limits. Although the minimum score for educational support was relatively low (55), it did not exceed the expected scale boundaries, and no extreme outliers were detected during data screening. Subscale means indicated moderate to high support for acquiring new knowledge (47.47 ± 6.81), concrete experience (39.72 ± 5.20), reflective observation (23.15 ± 3.37) and managerial support (22.76 ± 3.90). Resilience had a mean score of 38.53 (SD = 6.23), with a range of 18 to 50, indicating high resilience. The mean transition status was 149.27 (SD = 17.81), with a range of 96–190. The transition status subdimensions with the highest ratings were active coping strategies (40.38 ± 5.13) and interpersonal integration (39.37 ± 5.25), followed by competence (28.71 ± 3.72), positive emotion (27.54 ± 4.26) and work–life balance.

**TABLE 3:** Descriptive Statistics of educational support, resilience and transition status (*N* = 215).

Variable	(Mean ± SD)	Min–max
Educational support	133.11 ± 17.15	55–170
Acquiring new knowledge	47.47 ± 6.81	24–60
Concrete experience	39.72 ± 5.20	16–50
Reflective observation of own experience	23.15 ± 3.37	6–30
Support from home healthcare agency managers	22.76 ± 3.90	9–30
Resilience	28.58 ± 6.18	9–40
Transition status	149.27 ± 17.81	96–190
Interpersonal integration	39.37 ± 5.25	20–50
Active coping strategies	40.38 ± 5.13	19–50
Profession‐ related positive emotion	27.54 ± 4.26	13–35
Competence for nursing work	28.71 ± 3.72	14–35
Work–life balance	13.27 ± 3.90	4–20

As presented in Table 4, partial correlation analysis was conducted to examine the relationships among the main study variables after controlling relevant demographic and professional characteristics. Educational support remained significantly and positively correlated with resilience (partial *r* = 0.539, *p* < 0.001) and transition status (partial *r* = 0.639, *p* < 0.001). Resilience was also significantly and positively correlated with transition status (partial *r* = 0.614, *p* < 0.001). These findings suggest that the associations among educational support, resilience and transition status remained statistically significant after adjustment for potential covariates.

**TABLE 4:** Partial correlation matrix among the main study variables after controlling for demographic and professional characteristics (*N* = 215).

Variable		Educational support	Resilience	Transition status
Educational support	Partial *r*	1	0.539	0.639
	*p*‐value		< 0.001*	< 0.001*
Resilience	Partial *r*	0.539	1	0.614
	*p*‐value	< 0.001*		< 0.001*
Transition status	Partial *r*	0.639	0.614	1
	*p*‐value	< 0.001*	< 0.001*	

*Note:* Values represent partial correlation coefficients after controlling for age, gender, residence, income, marital status, experience in home healthcare, level of education, specialized training, number of patients cared for per week, number of working hours per week and type of home healthcare agency. All correlations were two‐tailed.

* *p* < 0.001.

Table **5** displayed the confirmatory factor analysis (CFA) model fit indices for the three study instruments. A second‐order CFA was conducted for the ESA‐NHHN and the TSSNGNs to evaluate whether their multidimensional structures could be represented by higher‐order constructs, whereas the CD‐RISC‐10 was evaluated using a one‐factor CFA model. The ESA‐NHHN demonstrated acceptable model fit (CFI = 0.949, TLI = 0.945, RMSEA = 0.079, SRMR = 0.059), supporting the use of the overall educational support score. The TSSNGNs showed marginal‐to‐acceptable model fit (CFI = 0.926, TLI = 0.921, RMSEA = 0.087, SRMR = 0.074), indicating qualified support for representing the five dimensions by an overarching transition status construct, although some fit indices remained below conventional good‐fit benchmarks. The CD‐RISC‐10 demonstrated marginal model fit as a one‐factor measure (CFI = 0.925, TLI = 0.904, RMSEA = 0.094), indicating that the proposed unidimensional structure showed mixed evidence of model fit in the present sample.

**TABLE 5:** Model Fit Indices for the Confirmatory Factor Analysis Models of the ESA‐NHHN, CD‐RISC‐10 and TSSNGNs.

Model	χ^2^	df	χ^2^/df	CFI	TLI	RMSEA	95% CI for RMSEA	SRMR	Interpretation
Second‐order four‐factor ESA‐NHHN model	1226	523	2.34	0.949	0.945	0.079	0.074–0.085	0.059	Acceptable fit
One‐factor CD‐RISC‐10 model	101	35	2.89	0.925	0.904	0.094	0.073–0.115	—	Marginal fit
Second‐order five‐factor TSSNGNs model	1720	660	2.61	0.926	0.921	0.087	0.082–0.092	0.074	Marginal‐to‐acceptable fit

***Note:*** Fit indices reported for the ESA‐NHHN and TSSNGNs are based on the scaled (robust) second‐order CFA models estimated using diagonally weighted least squares (DWLS) because the questionnaire items were ordinal. The ESA‐NHHN demonstrated acceptable model fit, whereas the TSSNGNs demonstrated marginal‐to‐acceptable fit. The CD‐RISC‐10 was evaluated using a one‐factor CFA model.

Abbreviations: χ^2^, chi‐square statistic; CFI, Comparative Fit Index; df, degrees of freedom; RMSEA, Root Mean Square Error of Approximation; SRMR, Standardized Root Mean Square Residual; TLI, Tucker–Lewis Index.

**FIGURE 1:**
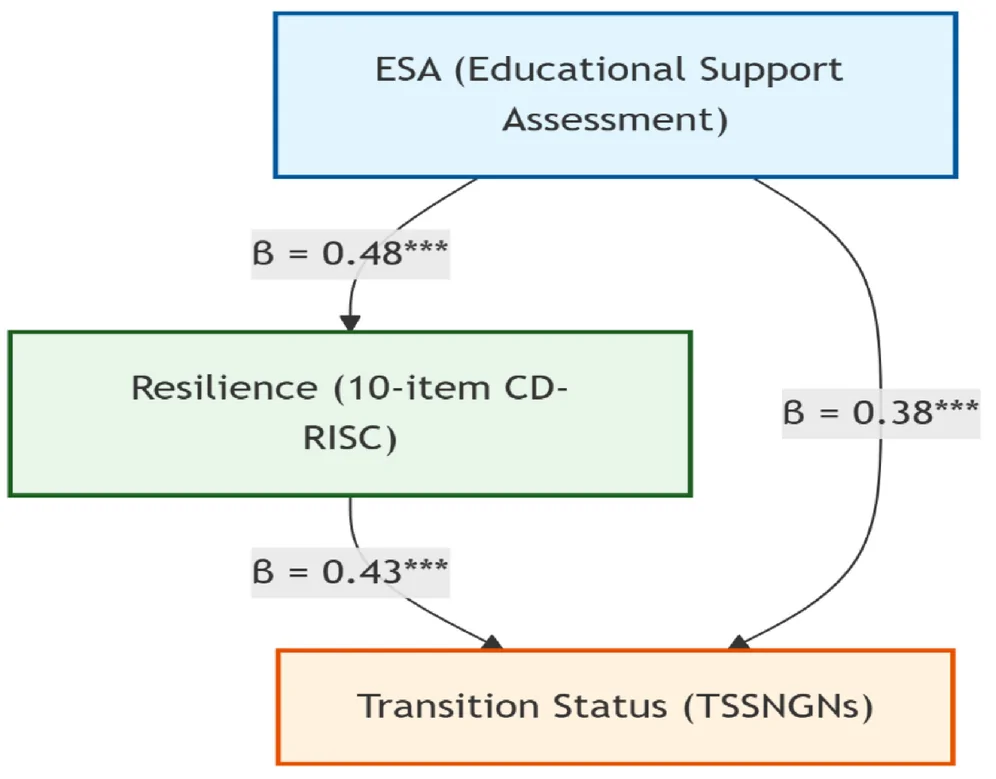
Structural Equation Model illustrates the direct and indirect effects of Educational Support Assessment (ESA) on Transition Status (TSSNGNs) through Resilience (10‐item CD‐RISC). Standardized path coefficients are presented. ESA = Educational Support Assessment; CD‐RISC‐10 = 10‐item Connor–Davidson Resilience Scale; TSSNGNs = Transition Status Scale for Newly Graduated Nurses; *β* = standardized path coefficient. The model was adjusted for sex, educational level, prior specialized training, average working hours per week and type of agency. Model fit: χ^2^(10) = 18.32, *p* = 0.049; CFI = Comparative Fit Index, 0.98; TLI = Tucker–Lewis Index, 0.96; RMSEA = Root Mean Square Error of Approximation, 0.06; SRMR = Standardized Root Mean Square Residual, 0.04. ***p < 0.001.

As shown in Table 6 and Figure 1, the structural equation model examined the standardized direct, indirect and total effects of Educational Support Assessment (ESA) on Transition Status (TSSNGNs) through Resilience (10‐item CD‐RISC), while adjusting for sex, educational level, prior specialized training, average working hours per week and type of agency. Educational support had a significant positive effect on resilience (*β* = 0.48, BootSE = 0.07, 95% BC bootstrap CI [0.36, 0.62]) and a significant direct effect on transition status (*β* = 0.38, BootSE = 0.08, 95% BC bootstrap CI [0.22, 0.52]). Resilience was also positively associated with transition status (*β* = 0.43, BootSE = 0.06, 95% BC bootstrap CI [0.29, 0.54]). In addition, educational support showed a significant indirect effect on transition status through resilience (*β* = 0.21, BootSE = 0.04, 95% BC bootstrap CI [0.13, 0.29]), resulting in a total standardized effect of *β* = 0.59 (BootSE = 0.07, 95% BC bootstrap CI [0.43, 0.69]). Because both the direct and indirect effects were statistically significant, the findings support a partial indirect association through resilience. The model demonstrated good overall fit, χ^2^(10) = 18.32, *p* = 0.049, CFI = 0.98, TLI = 0.96, RMSEA = 0.06 and SRMR = 0.04. Full covariate parameter estimates are provided in the Data S1.

**TABLE 6:** Standardized direct, indirect and total effects of educational support on transition status through resilience.

Effect type	Path	*β*	BootSE	95% BC bootstrap CI
Direct effect	ESA → transition status	0.38	0.08	0.22, 0.52
Indirect effect	ESA → resilience → transition status	0.21	0.04	0.13, 0.29
Total effect	ESA → transition status	0.59	0.07	0.43, 0.69
Path coefficient	ESA → resilience	0.48	0.07	0.36, 0.62
Path coefficient	Resilience → transition status	0.43	0.06	0.29, 0.54

*Note:* Standardized direct, indirect and total effects were estimated using Maximum Likelihood (ML). Indirect effects were evaluated using a nonparametric bootstrap procedure with 5000 bootstrap resamples, and bias‐corrected (BC) 95% confidence intervals were used to determine statistical significance. The model was adjusted for sex, educational level, prior specialized training, average working hours per week and type of agency. Full parameter estimates for the covariates are provided in the Data S1.

Abbreviations: BC, bias‐corrected; BootSE, bootstrap standard error; CD‐RISC, 10‐item Connor–Davidson Resilience Scale; CI, confidence interval; ESA, Educational Support Assessment; TSSNGNs, Transition Status Scale for Newly Graduated Nurses.

## 5 Discussion

The relationship between educational support and transition status was studied in novice home healthcare nurses, and whether resilience had an indirect relationship in this relationship was tested. The results confirmed the three hypotheses of the study. First, higher educational support was related to having a successful transition. Second, a positive relationship was found between education and resilience. Third, the transition status had a significant indirect association with educational support through resilience. These correlations were still present when controlling for other relevant demographic and occupational factors such as sex, education level, previous specialized training, mean work hours and home healthcare agency type.

The first prediction was confirmed: Novice home healthcare nurses who indicated higher levels of educational support indicated better transition status. The results of this finding align with transition to practice literature, indicating that structured support, preceptorship, clinical learning experiences and organizational guidance aid in the transition of newly graduated nurses to functioning in their roles (Arrowsmith et al. 2016; Graf et al. 2020; Ma et al. 2021; Baharum et al. 2023). Home health nurses may be the nurses with less immediate peer or supervisory support, with more complex patient and family situations and more autonomy in their practice, particularly in a home healthcare setting, which may make educational support more important. This discovery thus contributes to the existing body of knowledge on transition research by demonstrating that educational support is linked to positive transition outcomes in a novel, understudied setting: novice home healthcare nurses.

The second hypothesis was also supported, with educational support being positively correlated with resilience. The discovery implies that educational assistance might not be just related to professional preparation but also to the psychological resources required for adaptation. In the sense of educational support defined by Mori et al. (2023), it involves learning new content, gaining concrete experience, practicing and being supported by home healthcare agency managers. These supports can help to decrease uncertainty, increase perceived competence and aid in the processing of challenging clinical situations in a more positive light for novice nurses. This is aligned with the general occupational resilience literature, which defines resilience as an adaptive resource that supports maintaining or restoring effective functioning in response to stress, uncertainty, or adversity (Southwick et al. 2014; Robertson et al. 2015). Additionally, it aligns with the concept of psychological capital where resilience is viewed as a positive psychological resource linked with how an employee functions and his/her work‐related outcomes (Luthans et al. 2007).

The SEM supported the third hypothesis, as it showed a significant indirect relationship between educational support and transition status via resilience. There was a positive relationship between resilience and educational support, and a positive relationship between resilience and transition status. Meanwhile, there was still a direct pathway between educational support and transition status, suggesting a partial indirect pathway. This indicates that educational support has an association with transition status that is accounted for by resilience, but not all of the relationship. Educational support can be linked to the transition status in two ways: direct links, such as building knowledge and competence and preparing for roles and professional integration, and indirect links, such as providing learners with resilience as an adaptive capacity to deal with the demands of home healthcare practice.

This is a contribution to the adaptation literature for the workplace. Adjustment for a newcomer with an organization is considered a successful one when the person gets clarity of the role, self‐efficacy, social integration and adaptation in the work environment (Bauer et al. 2007; Saks and Gruman 2018). The present results indicate that novice home healthcare nurses' transition status is similar in terms of coping, competence building, interpersonal integration and adjusting to role demands. The findings are also in line with the Job Demands—Resources Model, which suggests that organizational resources, in combination with personal resources, facilitate the adjustment and work‐related outcomes of employees (Bakker and Demerouti 2007; Xanthopoulou et al. 2007). Educational support can be interpreted as an organizational resource, and resilience can be interpreted as a personal adaptive resource related to positive educational transition status.

Consistent with previous nursing research, the positive association between transition status and resilience was identified. Meyer and Shatto (2018) found that resilience was a factor in successful transition among new nurses and satisfaction with their jobs. Other more recent studies also suggest that resilience is correlated with lower levels of transition shock and greater retention‐related outcomes among new nurses (Xin et al. 2024). In the present study, we build on this evidence by examining the situation of novice home healthcare nurses in particular, who may be faced with unique transition demands as a result of their independent practice, home environments (which may be unpredictable), direct family involvement (which may be different) and limited immediate team support (which may be different).

The modified analyses support the main findings. In preliminary analyses, some demographic and professional variables differed, but the hypothesized associations were generally present when controlling for the covariates. Hence, it was not only the sex, level of education, previous specialized training, average working hours, or type of agency that explained the associations between educational support, resilience and transition status. This contributes to the importance of educational supports and resilience as key factors to understanding the transition status of novice HHCN.

In general, the results suggest that educational support is related to a positive transition status of novice home health nurses, and resilience is a significant adaptive influence in this relationship. The findings are relevant to the development of educational support programmes that are not exclusively technical but also include resilience‐building measures such as reflective supervision, structured feedback, peer discussion, stress‐management training and guided problem‐solving following challenging home visits. These strategies could help meet the clinical, psychological and contextual needs of beginning nurses as they move into the home healthcare setting.

### 5.1 Strengths and Limitations

There are several strengths in this study. It began by targeting novice home healthcare nurses, who are an under‐studied population whose transitions could be different from those of hospital‐based newly graduated nurses due to the early level of independence, the diversity of home environments, the nature of patient and family relationships and the absence of immediate supervision of this group of novice nurses. Second, the study employed a standardized set of instruments to assess aspects of education support, resilience and transition status. These tools were translated, culturally adapted, expert reviewed and pilot tested prior to data collection. Third, the analysis was strengthened by using adjusted partial correlations and structural equation modelling. The demographic and professional variables were controlled for to minimize the likelihood that the principal associations were simply due to these measured variables: sex, educational level, previous exposure to specialized training, average hours worked and type of agency.

However, there are certain weaknesses that need to be recognized. The cross‐sectional design does not allow for determining the temporal order of educational support, resilience and transition status. Hence, the indirect pathway is interpreted as a kind of associative statistical model and not as a causal pathway. In order to establish whether the educational support enhances the levels of resilience and transition status over time, more longitudinal and intervention studies would be required.

Secondly, it was a self‐reported study using questionnaires; recall bias, social desirability bias and common method variance may have affected the study. Additional data sources may be supervisor ratings, retention data, documented attendance at educational activities, etc., and should be explored in future studies. Third, the convenience sampling from the home healthcare agencies in one governorate may compromise the generalizability. Moreover, no information was collected on response rates at the agency level to determine whether agency‐level selection bias was added.

Fourth, even though several of the covariates measured were controlled, there may be residual confounding, as several factors, such as organizational culture, workload intensity, complexity of patients, quality of supervision, social support, family responsibilities, previous clinical experience and motivation for working longer hours, were not assessed. Fifth, some fit indices were marginal or sub‐optimal, which suggests a need for further validation of the Arabic versions of the instruments in larger and independent samples. Finally, other models could also be viable, such as a model suggesting that nurses with better transition status might experience more in terms of support, or that different aspects of resilience may affect experiences of and reporting of educational support.

## 6 Conclusions

This study explored the connection between educational support and the transition status of novice home healthcare nurses, as well as the indirect connection of resilience in this relationship. Educational support was positively related to transition status and resilience, and resilience was positively related to transition status. The SEM results also indicated that there was a significant indirect association of educational support with the transition status through the indirect association of resilience, in addition to the direct association.

The results indicated that educational support was a significant organizational resource related to novice home healthcare nurses' transition status. They also theorize that resilience is an adaptive psychological factor that is relevant in this relationship. Educational support can be especially useful in a home healthcare setting where novice nurses may be given early independence, different patient settings and less supervision in the immediate environment, allowing for an integration of clinical learning with techniques to promote resilience and adaptive coping.

## 7 Implications for Nursing Practice

From a nursing education perspective, these findings suggest that educational support should not focus only on clinical knowledge and technical skills. Home healthcare agencies may consider incorporating resilience‐focused components that require future evaluation. These may include structured preceptorship, guided reflection after difficult home visits, case‐based learning, stress‐management sessions, peer‐support groups and regular feedback meetings with supervisors. Nurse educators should also monitor novice nurses' adaptation during the first months of practice and provide early support for nurses who report low confidence, poor coping or work–life imbalance. Such strategies may help strengthen resilience and support better transition experiences among novice home healthcare nurses.

## Author Contributions

**Shaimaa Mohamed Amin:** conceptualization, writing – original draft, resources, supervision, data curation, project administration, validation, methodology, visualization, writing – review and editing, funding acquisition. **Heba Emad El‐Gazar:** conceptualization, formal analysis, software, writing – review and editing, writing – original draft. **Mohamed Ali Zoromba:** conceptualization, investigation, funding acquisition, writing – original draft, writing – review and editing, validation, software, formal analysis, data curation. **Heba Sobhy Mohamed:** conceptualization, writing – original draft, writing – review and editing, project administration, resources. **Mohammed Adel Abd Elhafeez Elbakry:** resources, data curation, project administration, writing – review and editing, writing – original draft, conceptualization. **Mohamed Ahmed Aly Mohamed:** conceptualization, writing – original draft, writing – review and editing, resources, project administration, methodology, investigation. **Mohamed Hussein Ramadan Atta:** conceptualization, investigation, writing – original draft, writing – review and editing, resources, visualization. **Sally Mohammed Farghaly Abdelaliem:** conceptualization, funding acquisition, writing – original draft, writing – review and editing, formal analysis, resources. **Nadiah A. Baghdadi:** resources, investigation, funding acquisition, conceptualization, writing – review and editing, writing – original draft. **Ayman Mohamed El‐Ashry:** conceptualization, investigation, methodology, validation, visualization, formal analysis, software, data curation, supervision, resources, writing – review and editing, writing – original draft.

## Funding

Princess Nourah bint Abdulrahman University Researchers Supporting Project number (PNURSP2026R293), Princess Nourah bint Abdulrahman University, Riyadh, Saudi Arabia.

## Disclosure

The authors confirm that the dataset used in this study has not been reported previously in another publication and that this manuscript represents an original analysis of the collected data.

## Ethics Statement

Ethical approval for this study was obtained from the Research Ethics Committee of the Faculty of Nursing, Zagazig University, Egypt, under reference number (295). In addition, permission to conduct the study was secured by clinic directors following a clear explanation of the study's purpose. The research strictly followed the ethical guidelines of the Declaration of Helsinki to uphold participants' rights and well‐being.

## Consent

All participants provided written informed consent after being fully briefed on the study's objectives.

## Conflicts of Interest

The authors declare no conflicts of interest.

## Supporting information


**Figure S1:** Second‐order confirmatory factor analysis model of the Educational Support Assessment Scale (ESA‐NHHN). Standardized factor loadings are displayed.
**Table S1:** Standardized loadings of the first‐order ESA‐NHHN dimensions on the second‐order Educational Support factor.
**Figure S2:** Second‐order confirmatory factor analysis model of the Transition Status Scale for Newly Graduated Nurses (TSSNGNs). Standardized parameter estimates are displayed.
**Table S2:** Standardized loadings of the first‐order TSSNGNs dimensions on the second‐order Transition Status factor.
**Table S3:** Standardized Covariate Effects in the Observed‐Variable Indirect‐Association Model.
**Table S4:** Confirmatory Factor Analysis for ESA‐NHHN.
**Table S5:** Confirmatory Factor Analysis for the transition status scale for newly graduated nurses (TSSNGNs).

## Data Availability

The dataset generated and/or analysed during the current study is not publicly available due to ethical and confidentiality considerations but may be obtained from the corresponding author upon reasonable request and subject to appropriate approval.
