# Supplementary material for: The Indirect Association of Resilience in the Relationship Between Educational Support and Transition Status Among Novice Home Healthcare Nurses
Source: Nurs Open. 2026 Sep 22;13(9):e70849. doi: 10.1002/nop2.70849 (PMC13597098; doi:10.1002/nop2.70849)
Supplement: Supplementary file 1 [file NOP2-13-e70849-s001.docx]

**Figure S1. Second-order confirmatory factor analysis model of the Educational Support Assessment Scale (ESA-NHHN). Standardized factor loadings are displayed.**

**Table S1. Standardized loadings of the first-order ESA-NHHN dimensions on the second-order Educational Support factor**

| First-order factor | Standardized loading (β) | p-value |
| --- | --- | --- |
| Acquire new knowledge | 0.831 | < .001 |
| Concrete experience | 0.983 | < .001 |
| Reflective observation of own experience | 0.973 | < .001 |
| Support from home healthcare agency managers | 0.846 | < .001 |

**Note.** All four first-order dimensions loaded significantly on the higher-order Educational Support factor. The strong standardized loadings support the representation of the ESA-NHHN dimensions by an overarching educational support construct.

Figure S2. Second-order confirmatory factor analysis model of the Transition Status Scale for Newly Graduated Nurses (TSSNGNs). Standardized parameter estimates are displayed

**Table S2. Standardized loadings of the first-order TSSNGNs dimensions on the second-order Transition Status factor**

| First-order factor | Standardized loading (β) | p-value |
| --- | --- | --- |
| Interpersonal Integration | 0.832 | < .001 |
| Active Coping Strategies | 0.867 | < .001 |
| Profession-Related Positive Emotion | 0.950 | < .001 |
| Competence for Nursing Work | 0.770 | < .001 |
| Work–Life Balance | 0.585 | < .001 |

**Note.** All five first-order dimensions loaded significantly on the higher-order Transition Status factor. The standardized higher-order loadings ranged from 0.585 to 0.950, supporting the representation of the five dimensions by an overarching transition status construct and the use of the overall composite score in the primary analyses.

**Table S3. Standardized Covariate Effects in the Observed-Variable Indirect-Association Model**

| Covariate path | β | SE | z | p | 95% CI |
| --- | --- | --- | --- | --- | --- |
| Prior specialized training → Resilience | 0.15 | 0.06 | 2.50 | .008 | [0.04, 0.26] |
| Prior specialized training → Transition Status | 0.17 | 0.06 | 2.83 | .005 | [0.05, 0.29] |
| Average working hours → Resilience | −0.09 | 0.05 | −1.80 | .070 | [−0.19, 0.01] |
| Average working hours → Transition Status | −0.11 | 0.05 | −2.20 | .030 | [−0.21, −0.01] |
| Educational level → Resilience | 0.05 | 0.05 | 1.00 | .340 | [−0.05, 0.15] |
| Educational level → Transition Status | 0.06 | 0.05 | 1.20 | .210 | [−0.04, 0.16] |
| Sex → Resilience | 0.02 | 0.06 | 0.33 | .720 | [−0.10, 0.14] |
| Sex → Transition Status | 0.04 | 0.05 | 0.80 | .380 | [−0.06, 0.14] |
| Type of agency → Resilience | −0.03 | 0.06 | −0.50 | .590 | [−0.15, 0.09] |
| Type of agency → Transition Status | 0.02 | 0.05 | 0.40 | .710 | [−0.08, 0.12] |

| Table S4. Confirmatory Factor Analysis for ESA-NHHN | | | | | |
| --- | --- | --- | --- | --- | --- |
| Factor | Indicator | Estimate | SE | Z | p |
| Acquire new knowledge | On entering employment, there is the opportunity to learn about the  system, including public healthcare insurance and government-based longterm care insurance | 0.512 | 0.0502 | 10.20 | <.001 |
|  | On entering employment, there is the opportunity to undergo training in  the compensation structure for home healthcare nursing and users’ responsibilities for fees | 0.573 | 0.0458 | 12.52 | <.001 |
|  | On entering employment, there is the opportunity to receive instruction on  the manners and conduct appropriate for home healthcare nurses | 0.570 | 0.0424 | 13.43 | <.001 |
|  | On entering employment, there is the opportunity to undergo training  about emergency responses and contact methods at patients’ homes. | 0.597 | 0.0480 | 12.44 | <.001 |
|  | On entering employment, there is the opportunity to learn about an overview of the workplace, appropriate policies, safety-related precautions, and  confirmation of the work schedule. | 0.548 | 0.0387 | 14.17 | <.001 |
|  | On entering employment, there is the opportunity to undergo training  about appropriate actions in the event of disasters. | 0.496 | 0.0398 | 12.45 | <.001 |
|  | On entering employment, there is the opportunity to receive information  about the location of the workplace, and local health, medical and welfare  resources | 0.488 | 0.0416 | 11.72 | <.001 |
|  | On entering employment, there is the opportunity to receive information  about the roles of other institutions and members of numerous professions | 0.513 | 0.0435 | 11.80 | <.001 |
|  | Within the workplace, there are regular opportunities for training and  participation in study groups | 0.605 | 0.0465 | 13.02 | <.001 |
|  | There are opportunities to undergo training for responding to users’ and  family members’ dissatisfaction and complaints | 0.542 | 0.0427 | 12.68 | <.001 |
|  | There are opportunities for training in taking the protection of users’ and  family members’ privacy into consideration when handling information and  records | 0.557 | 0.0447 | 12.45 | <.001 |
|  | Before being responsible for terminal-stage users, there is the opportunity  to undergo training about dealing with deaths at their homes. | 0.556 | 0.0506 | 10.99 | <.001 |
| Concrete experience | Experience can be gained in understanding the provision of care as a team,  together with colleagues and managers. | 0.383 | 0.0414 | 9.24 | <.001 |
|  | The 24-hour response system includes a mechanism for obtaining support  from colleagues promptly when needed | 0.486 | 0.0451 | 10.77 | <.001 |
|  | There are opportunities to understand that the consideration and manners  needed when visiting the homes of home-care patients are different from  those of a hospital nurse | 0.443 | 0.0410 | 10.81 | <.001 |
|  | One can repeatedly accompany colleagues on visits until one has gained  sufficient confidence. | 0.433 | 0.0498 | 8.70 | <.001 |
|  | There are opportunities to learn in practice how the status of the use of  sanitary materials differs between hospital and home healthcare nurses. | 0.498 | 0.0397 | 12.55 | <.001 |
|  | Newly appointed home healthcare nurses have opportunities to talk about  their own thoughts, feelings, and experiences and speak at meetings  for responsible personnel and so on, attended by members of multiple  professions | 0.580 | 0.0523 | 11.07 | <.001 |
|  | There are opportunities to appreciate the importance of information  exchange with members of other professions | 0.503 | 0.0449 | 11.21 | <.001 |
|  | There are opportunities to share one’s own experience of solo visits with  colleagues and others | 0.499 | 0.0505 | 9.88 | <.001 |
|  | There are places and environments that enable the provision of nursing  care that prioritizes the values and perspectives about the life and death of  home-care patients and their family members. | 0.559 | 0.0400 | 13.98 | <.001 |
|  | There are opportunities to learn about the importance of verbally expressing appreciation to family members who support home-care patients. | 0.528 | 0.0422 | 12.52 | <.001 |
| Reflective observation of own experience | At case-study meetings, there are opportunities to reflect on what one has  learned oneself and to integrate this with what other people have learned | 0.519 | 0.0387 | 13.39 | <.001 |
|  | There are opportunities to reflect on daily care, using checklists, and others,  together with one’s colleagues. | 0.505 | 0.0413 | 12.22 | <.001 |
|  | There are regular opportunities for face-to-face meetings with colleagues | 0.539 | 0.0449 | 12.00 | <.001 |
|  | There are opportunities to reflect on one’s own nursing home visits by  means of discussion with colleagues | 0.528 | 0.0437 | 12.08 | <.001 |
|  | There are opportunities to reflect on one’s own experience at conferences  and thus to fully appreciate the significance of that experience | 0.543 | 0.0456 | 11.89 | <.001 |
|  | There is support for setting targets for specific and realistic practice by  newly appointed home healthcare nurses. | 0.515 | 0.0401 | 12.86 | <.001 |
| Support from home healthcare agency managers | There is the potential to discuss with colleagues and/or managers the stress  felt in connection with home healthcare nursing activities. | 0.630 | 0.0504 | 12.51 | <.001 |
|  | Colleagues and/or managers form connections with newly appointed  home healthcare nurses to enable the nurses to develop a strong ability to  look carefully at and think about their daily experiences. | 0.598 | 0.0423 | 14.14 | <.001 |
|  | There are opportunities for regular face-to-face consultations with  managers. | 0.685 | 0.0451 | 15.20 | <.001 |
|  | Managers provide newly appointed home healthcare nurses feedback  about their work as a whole, as appropriate to the circumstances. | 0.625 | 0.0450 | 13.88 | <.001 |
|  | When newly appointed home healthcare nurses enter employment,  managers give them advice about ways to respond to problems with which  they have considerable experience | 0.557 | 0.0447 | 12.46 | <.001 |
|  | Managers respond on an individual level to newly appointed home health care nurses, taking each nurse’s background into consideration | 0.614 | 0.0448 | 13.70 | <.001 |

**Table S5: Confirmatory Factor Analysis for the transition status scale for newly graduated nurses (TSSNGNs)**

| Factor | Indicator | Estimate | SE | Z | p |
| --- | --- | --- | --- | --- | --- |
| Interpersonal integration | My colleagues treat me very sincerely | 0.475 | 0.0566 | 8.40 | <.001 |
|  | When I encounter difficulties, my colleagues will be willing to help me | 0.511 | 0.0427 | 11.96 | <.001 |
|  | I have a harmonious relationship with my colleagues | 0.456 | 0.0367 | 12.44 | <.001 |
|  | My colleagues give me enough respect | 0.516 | 0.0391 | 13.21 | <.001 |
|  | I feel relaxed when I get along with colleagues | 0.533 | 0.0416 | 12.80 | <.001 |
|  | I have a sense of belonging to my work unit | 0.457 | 0.0515 | 8.87 | <.001 |
|  | I feel accepted by my colleagues | 0.491 | 0.0340 | 14.44 | <.001 |
|  | My colleagues can understand me | 0.486 | 0.0376 | 12.93 | <.001 |
|  | I consider my work unit is a warm family | 0.532 | 0.0492 | 10.81 | <.001 |
|  | I communicate smoothly with leaders, and colleagues | 0.469 | 0.0575 | 8.16 | <.001 |
| Active coping strategies | I can identify the factors preventing me from achieving my goals. | 0.472 | 0.0401 | 11.79 | <.001 |
|  | I know what kind of person I am | 0.495 | 0.0386 | 12.83 | <.001 |
|  | When I encounter difficulties, I will find a way to solve them actively. | 0.520 | 0.0420 | 12.40 | <.001 |
|  | I can recognize my strengths and weaknesses | 0.433 | 0.0364 | 11.90 | <.001 |
|  | I can actively seek resources and help when encountering problems | 0.457 | 0.0378 | 12.10 | <.001 |
|  | I know what resources I can use to achieve my goals | 0.447 | 0.0414 | 10.78 | <.001 |
|  | In order to reach my goal, I have a clear plan of action | 0.532 | 0.0409 | 13.01 | <.001 |
|  | I can overcome the factors that prevent me from achieving my goals | 0.509 | 0.0416 | 12.24 | <.001 |
|  | I often reflect on my work. | 0.487 | 0.0413 | 11.80 | <.001 |
|  | I believe that the problem can be solved through my hard work. | 0.551 | 0.0453 | 12.18 | <.001 |
| Profession-related positive emotion | I am willing to be a nurse | 0.608 | 0.0577 | 10.53 | <.001 |
|  | I am interested in the work I do | 0.420 | 0.0414 | 10.15 | <.001 |
|  | I am full of hope for the future | 0.555 | 0.0488 | 11.36 | <.001 |
|  | I am satisfied with my current state. | 0.603 | 0.0590 | 10.22 | <.001 |
|  | I have a sense of accomplishment in my work | 0.576 | 0.0426 | 13.52 | <.001 |
|  | When I think I am going to work tomorrow, I feel calm in my mind | 0.620 | 0.0662 | 9.37 | <.001 |
|  | I have clear career development goals | 0.550 | 0.0459 | 11.97 | <.001 |
| Competence for nursing work | I can provide care for patients independently | 0.494 | 0.0386 | 12.80 | <.001 |
|  | I can meet the assessment standards of the hospital | 0.532 | 0.0424 | 12.55 | <.001 |
|  | I know quite well about my daily work content and procedures | 0.529 | 0.0360 | 14.69 | <.001 |
|  | I feel competent in my work. | 0.525 | 0.0380 | 13.80 | <.001 |
|  | I can do my nursing work in an orderly way | 0.491 | 0.0358 | 13.71 | <.001 |
|  | I can detect changes in the patient's condition in time | 0.494 | 0.0407 | 12.14 | <.001 |
|  | I am able to communicate about patients' condition with the  healthcare team | 0.486 | 0.0373 | 13.03 | <.001 |
| Balance between work and life | My work does not prevent me from achieving relaxation in my life | 0.807 | 0.0652 | 12.38 | <.001 |
|  | I will not lose sleep because of work | 1.043 | 0.0658 | 15.85 | <.001 |
|  | I have time to do what I want to do after work | 0.953 | 0.0631 | 15.12 | <.001 |
|  | Overall, I think I can achieve a balance between work and life | 0.896 | 0.0588 | 15.23 | <.001 |
